# Endometriosis development in relation to hypoxia: a murine model study

**DOI:** 10.1186/s10020-024-00973-x

**Published:** 2024-10-30

**Authors:** Marta Hoffmann-Młodzianowska, Radosław B. Maksym, Katarzyna Pucia, Monika Kuciak, Andrzej Mackiewicz, Claudine Kieda

**Affiliations:** 1 Laboratory of Molecular Oncology and Innovative Therapies, Military Institute of Medicine - National Research Institute, 04-141 Warsaw, Poland; 2 1st Department of Obstetrics and Gynaecology, Centre of Postgraduate Medical Education, 01-004 Warsaw, Poland; 3 Animal Experimentation Laboratory of the Center for Biostructure Research, Medical University of Warsaw, 02-106 Warsaw, Poland; 4 Chair of Medical Biotechnology, Poznan University of Medical Sciences, 61-806 Poznan, Poland; 5 Department of Diagnostics and Cancer Immunology, Greater Poland Cancer Centre, 61-866 Poznan, Poland; 6 Centre for Molecular Biophysics, UPR4301 CNRS, 45071 Orléans, France

**Keywords:** Endometriosis, Murine model of endometriosis, Hypoxia, PTEN, EF5, High-resolution ultrasound

## Abstract

**Background:**

Endometriosis, due to its ambiguous symptoms, still remains one of the most difficult female diseases to treat, with an average diagnosis time of 7–9 years. The changing level of hypoxia plays an important role in a healthy endometrium during menstruation and an elevated expression of the hypoxia-inducible factor 1-alpha (HIF-1α) has been demonstrated in ectopic endometria. HIF-1α mediates the induction of proangiogenic factors and the development of angiogenesis is a critical step in the establishment and pathogenesis of endometriosis. Although the inhibition of angiogenesis has been proposed as one of the actionable therapeutic modalities, vascular normalization and re-oxygenation may become a possible new approach for therapeutic intervention.

**Methods:**

Our goal was to investigate whether a selected murine model of endometriosis would be suitable for future studies on new methods for treating endometriosis. Non-invasive, high-resolution ultrasound-monitored observation was selected as the preclinical approach to obtain imaging of the presence and volume of the endometriotic-like lesions. The EF5 (2-(2-Nitro-1H-imidazol-1-yl)-N-(2,2,3,3,3-pentafluoropropyl)acetamide) compound that selectively binds to reduced proteins in hypoxic cells was used for hypoxia detection. The expression of *Pten* and other crucial genes linking endometriosis and hypoxia were also assessed.

**Results:**

Using EF5, a pentafluorinated derivative of the 2-nitroimidazole that is metabolically reduced by oxygen-inhibitable nitroreductase, we confirmed that hypoxia did develop in the selected model and was detected in uterine and ectopic endometriotic lesions. Moreover, the changes in oxygen tension also influenced the expression level of significant genes related to endometriosis, like *Pten*, *Trp53*, *Hif1a*, *Epas1*, and *Vegfa*. Their strong modulation evidenced here is indicative of model reliability. Using high-resolution ultrasound-based imaging, we present a non-invasive method of visualization that enables the detection and observation of lesion evolution throughout the duration of the experiment, which is fundamental for further preclinical studies and treatment evaluation.

**Conclusions:**

The selected model and method of visualization appear to be suitable for the study of new treatment strategies based on hypoxia alleviation and blood flow restoration.

## Introduction

Cells and tissues need sufficient oxygen availability to maintain proper homeostasis. It is well known that an imbalance between tissue oxygen supply and consumption is a characteristic feature of various diseases, including those of the reproductive system. Endometriosis is a chronic and inflammatory gynaecological disease characterized by the presence of endometrial-like tissue located outside the uterine cavity. It is reported that endometriotic lesions in women present increased levels of hypoxia (Li et al. 2021). Hypoxia is a state in which oxygen tension is much lower than under physiological conditions, called physioxia, which itself reflects the oxygen tension actually occurring in various parts of the body, from 13% in the oxygenated blood of the lungs, to 1% in the skin dermoepidermal junction, hair follicles, and sebaceous glands (Carreau et al. 2011). Ambient conditions (21% O_2_) equilibrate to the classical cell culture medium at around 18.5% O_2_. Hypoxia can vary in intensity, from mild where the tension of oxygen oscillates between 1 and 5%, to severe, below 1% (Koh and Powis 2012). The oxygen shortage leads to the activation of transcription factors, termed as hypoxia-inducible factors (HIFs). An ectopic endometrium demonstrates an elevated expression of the hypoxia-inducible factor 1-alpha (HIF-1α), in comparison to a eutopic endometrium (Wu et al. 2007). HIF-1α mediates the induction of proangiogenic factors, such as vascular endothelial growth factor (VEGF), an overexpression of which has been observed in the peritoneal fluid of women suffering from endometriosis (Donnez et al. 1998; McLaren et al. 1996). VEGF and chronic inflammation are strong regulators of angiogenesis development, which is a critical step in the establishment and the pathogenesis of endometriosis (Taylor et al. 2002). Although the inhibition of angiogenesis was proposed as one of the actionable therapeutic modalities, it appears to generate adverse effects due to the resulting exacerbated hypoxia (Klauber et al. 1997). This raises the concept of vascular normalization to stabilize the properly formed new vessels, which may become a possible new approach for therapeutic intervention (Jain 2005; Aplin and Nicosia 2023). Vessel normalization and the re-oxygenation of hypoxic sites may allow optimal therapeutic efficacy to be achieved. This has already been demonstrated in radiotherapy that relies on adequate tissue oxygenation or chemotherapy, which depends on efficient tumour blood flow and, therefore, drug delivery to the treated cells (Goel et al. 2011). The effectiveness of such a strategy has been shown in cancer treatment (Kieda et al. 2012), where the normalisation of blood vessels by alleviating hypoxia resulting in the phosphatase and tensin homolog (PTEN) activation has been demonstrated to be key in the treatment of hypoxia-related diseases, such as cancer. Indeed, angiogenesis normalization, induced by the alleviation of hypoxia, deeply modifies the tumour microenvironment and reverts immunosuppression to an antitumor immune response (Hafny-Rahbi et al. 2021).

PTEN, a tumour suppressor gene that is a key negative regulator of the phosphatidylinositol 3-kinase (PI3K) pathway, is one of the factors that control angiogenesis. It has been shown that the PTEN protein level is controlled by hypoxia and that the downregulation of the PTEN gene results in the accumulation of HIF-1α (Majewska et al. 2021; Nascimento-Filho et al. 2019). PTEN mutations have been identified in endometrial hyperplasias, as well as in carcinomas, which suggests that the level of PTEN gene expression is important in pathologies related to the female reproductive system (Maxwell et al. 1998; Salvesen et al. 2002). PTEN is involved in the regulation of focal adhesion, cellular migration, and tumour cell proliferation, and its inactivation can lead to a loss of its tumour suppressor function (Li et al. 1997; Tamura et al. 1998). The reoxygenation of hypoxic tumour cells, which results in the selective activation of PTEN, restores healthy vasculature and offers new treatment possibilities (Kieda et al. 2012). Moreover, it was suggested that a positive staining of PTEN in patients with an advanced endometrial carcinoma is a significant prognostic indicator of favourable survival. Thus, PTEN expression analysis may contribute to the individualization of treatment (Kanamori et al. 2002). The presented data indicate that reversing hypoxia and vessel normalization may be a novel approach to the treatment of endometriosis.

Endometriosis affects approximately 10% of women of reproductive age and usually manifests itself through pelvic pain and reduced fertility (Zondervan et al. 2020). According to the latest data, the average time of endometriosis diagnosis is 7–9 years globally (Ghai et al. 2020). As a disease that lasts for decades and affects many spheres of a patient’s life, it requires a multi-specialist approach and a long-term treatment strategy. The treatment or alleviation of the effects of endometriosis is usually tailored to the condition and needs of the patients, but there is still an absence of an accessible, effective, and safe method of treatment and prevention of the recurrence of endometriosis. Therefore, finding new therapies continues to pose enormous challenges.

The mouse, as a molecularly well-annotated species, provides a powerful model in endometriosis research. Since rodents cannot spontaneously form ectopic lesions, endometriosis-like lesions are induced by transplanting endometrial tissue to ectopic sites (Laschke and Menger 2007; Laschke et al. 2008). A large number of murine models of endometriosis have been described thus far (Burns et al. 2022). In the presented study, we have recreated a murine model of endometriosis by suturing uterine tissue samples to the peritoneal wall. Our goal was to investigate whether the selected model would be suitable for future studies on new treatment methods of endometriosis by reversing on-site hypoxia. This involves the detection and observation of the evolution of the lesions by a non-invasive, stress-free method applied to living animals, thus, minimizing the number of animals in the study is crucial to such experiments. Therefore, a non-invasive, high-resolution ultrasound-monitored observation was selected as the preclinical approach to obtain imaging of the presence and volume of the endometriotic-like lesions. The EF5 (2-(2-Nitro-1H-imidazol-1-yl)-N-(2,2,3,3,3-pentafluoropropyl)acetamide) compound that selectively binds to reduced proteins in hypoxic cells was used for hypoxia detection. EF5, through its lipophilic and uncharged structure, distributes very rapidly and evenly in tissues (Koch 2002). Finally, the expression of *Pten* and other crucial genes linking endometriosis and hypoxia were also assessed.

## Materials and methods

### Animals

For the purposes of the study, 6–8 weeks old female BALB/c mice were used. All the animal work was performed in the animal facility at the Animal Experimentation Laboratory of the Center for Biostructure Research at the Medical University of Warsaw. All the experiments were approved by the local ethics committee (approval number WAW2/155/2021, WAW2/141/2022) and were conducted in accordance with local legislation (Polish Act on the Protection of Animals Used for Scientific or Educational Purposes). The animal density and maintenance were in accordance with the Directive 2010/63/EU of the European Parliament and of the Council of 22 September 2010 on the protection of animals used for scientific purposes. All mice were on a 12:12 light–dark cycle (air temperature 20–24 °C, humidity 55% ± 10%) with ad libitum access to food (pellet) and water. Animals had access to environmental enrichments (e.g. hiding places, nesting material, aspen chew bricks).

### Model of endometriosis

The oestrous cycle in mice was synchronized by the modified Whitten effect, where female mice were exposed to male pheromones from the soiled bedding transferred from male mice cages (Dalal et al. 2001). To verify the stage of the oestrous cycle, murine external genitalia were visually evaluated (Byers et al. 2012). The synchronized mice were randomly divided into two groups: the control group (n = 5), and the endometriosis-induced group (ENDO group, n = 5). Endometriosis in mice was surgically induced, as previously described, with minor modifications (Cummings and Metcalf 1995; Körbel et al. 2010). Briefly, the abdominal wall of the anesthetized mouse was open by median laparotomy. One of the uterine horns was excised and transferred into a Petri dish containing cold, sterile phosphate-buffered saline (PBS) (#14190250, Thermo Fisher Scientific Inc., Waltham, MA, USA). The uterine horn was opened longitudinally, and tissue samples were removed using a 3 mm Keyes biopsy punch (#HH.510.030, Falcon, Lodz, Poland). Tissue samples were sutured to the peritoneal wall with a 5/0 Vicryl Rapide suture (#V4930H, Johnson & Johnson, New Brunswick, NJ, USA), two on the right, and two on the left side of the anterior abdominal wall, approximately 8 mm laterally from the median incision. The implants were attached in such a way that the inner surface of the developed uterus (the surface of the endometrium) was stitched to the surface of the peritoneum. The tissue samples were about 1 cm apart in the caudal-cranial axis. Mice from the control group were sham operated: a median laparotomy was performed, one uterine horn was ligated and excised, but no lesion was implanted. All the surgical procedures and imaging were performed under anaesthesia, by injection of a ketamine/xylazine solution (80 mg/kg b wt/10 mg/kg b wt; Vetoquinol, Lure Cedex, France; Biowet Pulawy, Pulawy, Poland), and the mice were observed until fully recovered. On day 21 of the experiment, the mice were sacrificed for tissue collection.

### High-resolution ultrasound imaging

The developed endometriotic lesions in mice were observed at days 10 and 21 using Ultra High-Frequency ultrasound—the Vevo 3100 and MX550D transducer (FujiFilm VisualSonics, Toronto, ON, Canada). The ultrasound imaging included a determination of the presence of endometriotic lesions in the mice from the ENDO group and colour Doppler imaging was used to detect the presence of the surrounding vessels. The images were analysed using Vevo LAB software (FujiFilm VisualSonics), and this included measurements of the size and 3D volume of the lesion created by acquiring a series of 2D slices and assembling them into a 3D data set, with a 0.15 mm step size between the slices.

### Tissue hypoxia quantification and level

To determine the hypoxia level, the mice were intraperitoneally injected with the EF5 (2-(2-Nitro-1H-imidazol-1-yl)-N-(2,2,3,3,3-pentafluoropropyl)acetamide) hypoxia-detecting agent, 2 h before euthanasia (#EF5-30A4, Merck, Darmstadt, Germany) for adduct formation with the reduced macromolecules in hypoxic cells. The collected tissues were frozen, sectioned, fixed, and stained with Alexa Fluor 488 conjugated antibodies ELK3-51 raised against EF5 adducts, according to the manufacturer’s instructions (#EF5-30A4, Merck). The nuclei were stained with 4′6-diamidino-2-phenylindole—DAPI (#P36935, ProLong Gold Antifade Mountant, Thermo Fisher Scientific Inc.). Tissue imaging was collected using the Zeiss Axio Observer with Axiocam 503 mono and Apotome 3 (Zeiss, Oberkochen, Germany). The mean value of the relative fluorescence intensity was quantified using ZEN Microscopy Software (Zeiss). The fluorescence from the epithelial and stromal cells was measured in the uteri from the control group (n = 3), from the group with induced endometriosis (n = 3), and from the group with endometriotic lesions (n = 2), in three repetitions. The mean staining intensity value for Regular Stain (stained with antibodies ELK3-51 raised against EF5) and No Stain (without antibodies ELK3-51 against EF5) was calculated as follows: Alexa Fluor 488-ELK3-51/DAPI. The final mean staining intensity values have been shown as a Regular Stain/No Stain ratio.

### Total RNA isolation and real‐time PCR analysis

The total RNA from the collected tissues was isolated using Direct-zol RNA MiniPrep (#R2052, Zymo Research, Irvine, CA, USA), according to the manufacturer’s instructions. The RNA samples were, *in-column* DNase I, treated to remove genomic DNA contamination. The RNA quality and concentrations were evaluated using Thermo Scientific μDrop Plate and Thermo Scientific SkanIt software (#N12391, Thermo Fisher Scientific Inc.). cDNA was synthesized using a High-Capacity cDNA Reverse Transcription Kit with RNase Inhibitor (#4374966, Applied Biosystems, Waltham, Massachusetts, USA). The resulting cDNA preparations were analysed using CFX Connect Real-Time System and CFX Maestro software (Bio-Rad, Hercules, CA, USA), performed with TaqMan Gene Expression Assays in combination with TaqMan Gene Expression Master Mix (#4369016, Applied Biosystems). The thermal cycling conditions were as follows: 50 °C for 2 min, 95 °C for 10 min, followed by 40 cycles of 95 °C for 15 s, and 60 °C for 60 s. The following TaqMan gene expression assays were used: *Pten* (Mm00477208_m1), *Trp53* (Mm01731287_m1), *Mdm2* (Mm01233138_m1), *Hif1a* (Mm00468869_m1), *Epas1* (Mm01236112_m1), and *Vegfa* (Mm00437306_m1). Expression levels were normalized against the endogenous control gene *Rpl7* (Mm02342562_gH). The relative expression was quantified using the 2^−ΔΔCt^ method (Livak and Schmittgen 2001). PCR analysis was performed on ≤ 3 animals in three repetitions.

### pO_2_ measurements

Tissue oxygen partial pressure (pO_2_) was measured using the OxyLite Pro instrument (Oxford Optronix Ltd., Adderbury, UK). This device measures the dissolved oxygen in tissues or fluid by determining the fluorescence light that is quenched proportionally to the pO2 level in the vicinity of the dye. The sensing tip contains a platinum-based fluorescent dye held within a polymer matrix. The sensor tip (NX-LAS-1/OT/E, Oxford Optronix Ltd.) was placed inside the uterine and endometriotic lesions (the cyst lumen and wall of the endometriotic lesion) immediately before tissue collection. The pO_2_ signal was recorded until the signal was stable. Due to the limitation of the animals in this pilot study, the small size of the organs, the fragile structure and preciousness of the examined tissues, the measurements were performed on one representative animal.

### Statistics

The data are presented as the mean ± SEM of three independent experiments performed in triplicate. The Shapiro–Wilk test was used to test the normality of data distribution. Statistical analysis for normally distributed data between the two groups (EF5 staining) was performed using the parametric t test. The non-normally distributed data between the three groups (PCR analysis) was tested using the one-way ANOVA (Kruskal–Wallis) test. The level of significance was set at *P* < 0.05. Statistical analyses were performed using the GraphPad Prism Software (San Diego, CA, USA).

## Results

### Imaging of endometriotic lesions developed in a mouse model of endometriosis

**Fig. 1:**
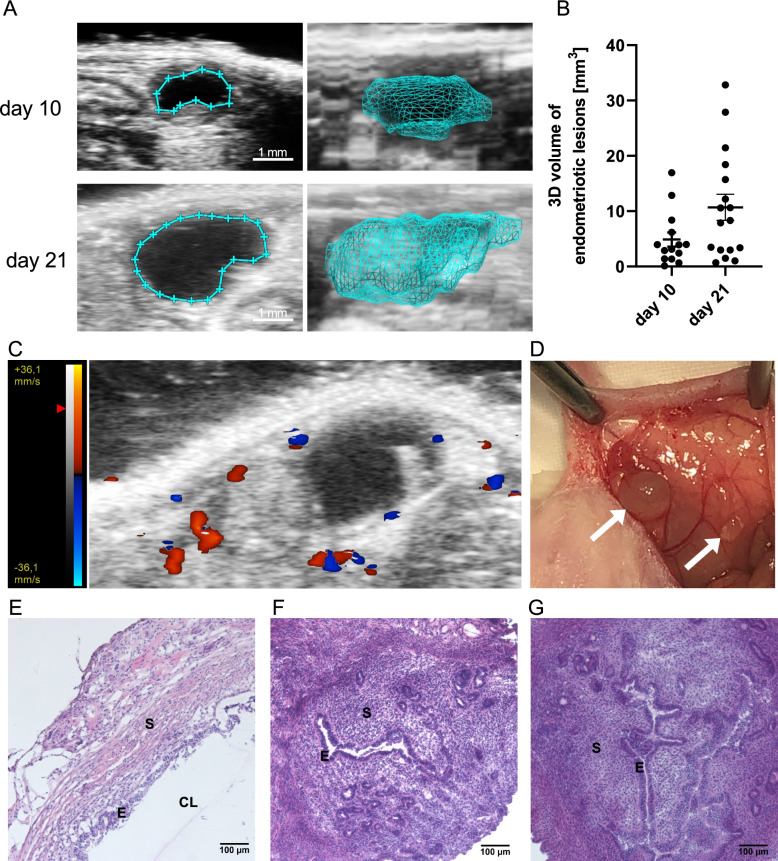
High-resolution ultrasound 2D imaging of a developed endometriotic lesion (borders marked by the blue line) at days 10 and 21; 3D reconstruction of an endometriotic lesion created by acquiring a series of 2D slices and assembling them into a 3D data set (step size between slices: 0.15 mm. Scale bar: 1 mm, (**A**). Average 3D volume distribution of endometriotic lesions developed in mice (n = 5) on days 10 and 21 (**B**). Presence of a vessel around lesions has been visualized using colour Doppler mode (the blue colour indicates blood flow away from the transducer; the red colour indicates blood flow towards the transducer, (**C**). Example of developed endometriotic lesions (caudal and cranial) as fluid-filled cysts at day 21 of the experiment (white arrows, **D**). Histological hematoxylin/eosin staining of the endometriotic lesion (fragment of the endometriotic lesion wall, **E**), the uterus from the mouse with the induced endometriosis (ENDO group, **F**), and the uterus from the control mouse (control group, **G**), with the location of the cyst lumen marked as CL, the epithelial cells as E, and the stroma as S. Scale bars: 100 µm

According to previously published data (Cummings and Metcalf 1995; Körbel et al. 2010), suturing tissue samples obtained from a mouse uterine horn to a peritoneal wall enables the creation of implants imitating endometriotic-like lesions in mice. In the present study, on days 10 and 21 of the experiment, ultrasound imaging detected the development of endometriotic cystic lesions, which were observed in all the mice from the ENDO group. This non-invasive method of visualization by acquiring a series of 2D slices allowed them to be assembled into a 3D data set to measure the 3D volume of the developed endometriotic lesions (Fig. 1A). The mean 3D volume of the developed lesions was 4.87 mm^3^ (n = 14) and 10.70 mm^3^ (n = 17) on days 10 and 21, respectively (Fig. 1B). Majority of the induced endometriotic lesions were observed at day 21. Endometriotic lesions that were difficult to localized by ultrasound visualization were observed during tissue collection. The blood flow through the forming blood vessels was observed using Colour Doppler mode (Fig. 1C). Developing endometriotic lesions and small surrounding blood vessels were also observed during tissue collection (Fig. 1D). The histological examination of the tissue samples demonstrated that uterine grafts developed typical endometriotic-like structures: an epithelial layer surrounding the cyst lumen and endometrial stroma (Fig. 1E). Figure 1G and H shows hematoxylin/eosin staining of the uterus from the mouse with the induced endometriosis (Fig. 1F), and the uterus from the control mouse (Fig. 1G). The images obtained have evidenced the possibility of the model of endometriosis being recreated in mice. The next step involved investigating whether hypoxia would be observed in the collected tissues.

### Assessment of hypoxia in uterine and endometriotic lesions using hypoxia-detecting molecule, EF5

**Fig. 2:**
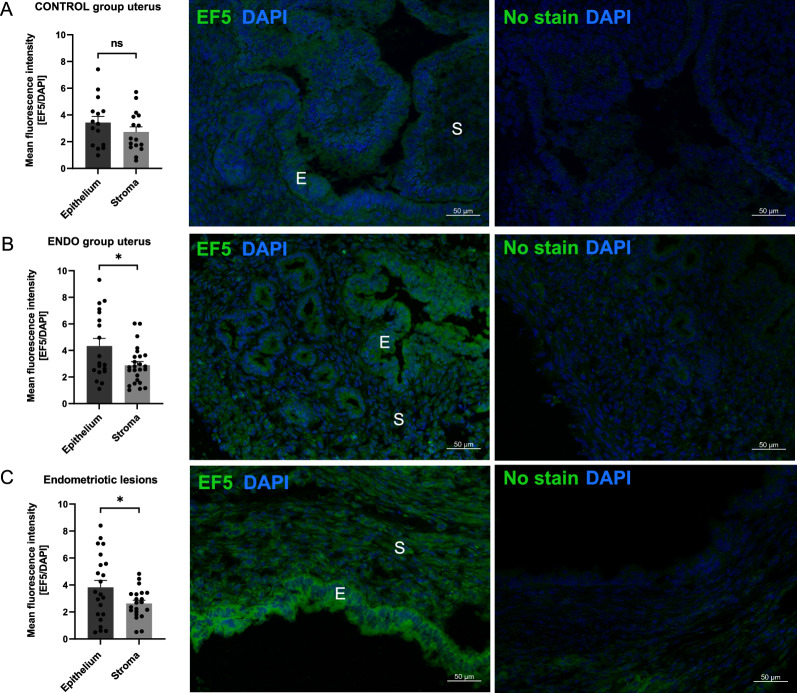
Analysis of tissue hypoxia using hypoxia-detecting molecule, EF5. Mean fluorescence intensity value in epithelial cells and stromal cells were measured and compared in the uteri of the control mice (control group, **A**), the uteri from mice with the induced endometriosis (ENDO group, **B**), and with the endometriotic lesions (**C**), epithelial cells—E, and stroma—S. Scale bars: 50 µm. *P < 0.05, no significance—ns

The tissue hypoxia level was measured using the EF5 compound that selectively binds to hypoxic cells and forms adducts. No statistical significance was observed in the hypoxia levels, reported as the relative mean fluorescence intensity, between the epithelial and stromal cells in the control uterus (Fig. 2A). The hypoxia level in the epithelial layer surrounding the cyst lumen in the uterus from the group with the induced endometriosis was higher than in the stromal cells (Fig. 2B, 4.33 ± 0.57 vs 2.89 ± 0.28, respectively). Similarly, the hypoxia level in the epithelial layer was higher than in the stromal cells in the endometriotic lesions (Fig. 2C, 3.82 ± 0.52 vs 2.63 ± 0.25, respectively). Upon finding that the tissue hypoxia level changes in samples collected from mice with the induced endometriosis, we investigated if such changes would also be observed on gene expression level.

### Basal Pten, Trp53, Mdm2, Hif1α, Epas1, and Vegfa gene expression in uterine and endometriotic lesions

**Fig. 3:**
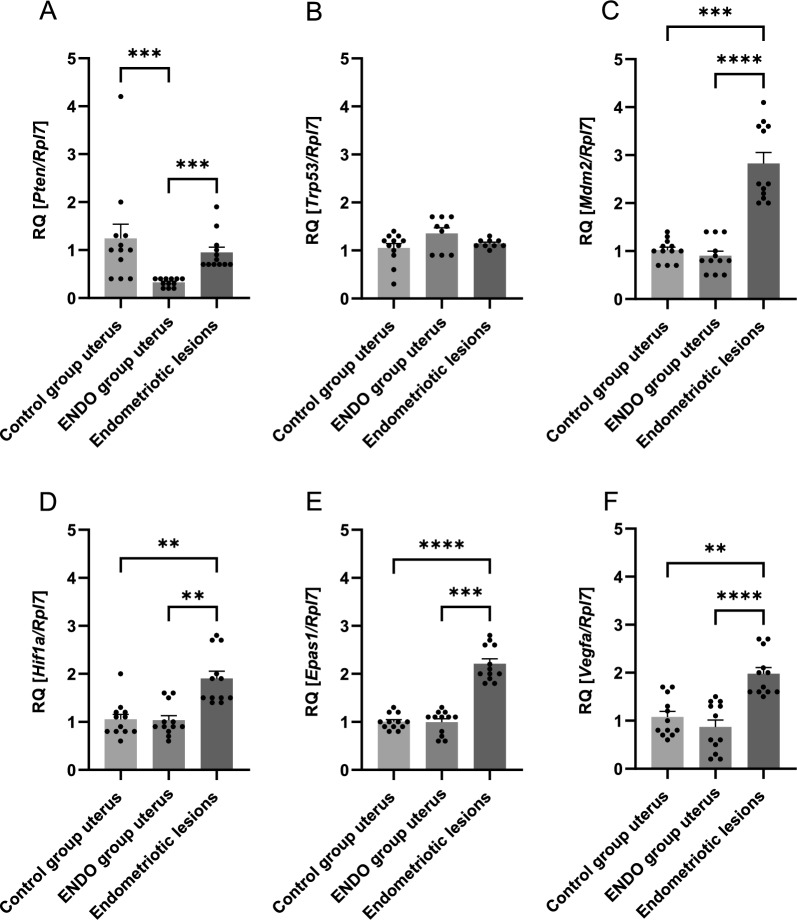
Basal mRNA expression of *Pten* (**A**), *Trp53* (**B**), *Mdm2* (**C**), *Hif1a* (**D**), *Epas1* (**E**), and *Vegfa* (**F**) in the control group uteri vs the ENDO group uteri vs the endometriotic lesions (**P ≤ 0.003; ***P ≤ 0.0003; ****P < 0.0001). RQ, relative quantity

PCR was performed to determine whether the changes in oxygen tension also influenced the expression level of significant genes related to endometriosis and hypoxia. We quantified the basal expression levels of the phosphatase and tensin homolog (*Pten*), transformation-related protein 53 (*Trp53*), transformed mouse 3T3 cell double minute 2 (*Mdm2*), hypoxia-inducible factor 1, alpha subunit (*Hif1α*), endothelial PAS domain protein 1 (*Epas1,* also known as hypoxia-inducible factor-2 alpha), and vascular endothelial growth factor A (*Vegfa*) in the uterus from the control group, the ENDO group, and the group with endometriotic lesions by real-time PCR. For each gene, the relative quantity (RQ) was generated by the control group uteri in Fig. 3A–F.

The basal *Pten* transcript level for the ENDO group uteri decreased and was 0.3 RQ as compared to the control group uteri (Fig. 3A). The basal expression levels of *Trp53, Mdm2, Hif1a, Epas1,* and *Vegfa* were similar among the control and the ENDO group uteri (Fig. 3B–F). The next step involved comparing the selected gene expressions in the endometriotic lesions to the control and the ENDO group uteri. The *Pten* and *Trp53* gene expression in the endometriotic lesions was similar when compared to the control group uteri. Whereas *Mdm2*, *Hif1a*, *Epas1,* and *Vegfa* expression increased in endometriotic lesions (2.8-, 1.9-, 2.2- and 2.0-fold, respectively, Fig. 3C–F). When compared to the ENDO group uteri, the expression of *Pten*, *Mdm2*, *Hif1a*, *Epas1,* and *Vegfa* was higher in the endometriotic lesion group (2.7-, 3.1-, 1.8-, 2.2-, and 2.3-fold, respectively, Fig. 3A, C–F). Transcript level for *Trp53* showed no changes (Fig. 3B). These results demonstrated that changes in endometriotic lesions are present in terms of the expression of genes associated with hypoxia when compared to control or ENDO group uteri.

### Oxygen tension in uterine and endometriotic lesions

In order to obtain as much data as possible on the presence of hypoxia in endometrial lesions, measurements of the partial oxygen pressure using the OxyLite device were performed. In the control group uteri, the pO_2_ level was 14 mmHg; whereas, in the cyst lumen and wall of the endometriotic lesion, the pO_2_ level was 7 mmHg and 2.4 mmHg, respectively. This observation supports the fact that oxygen partial pressure decreased in endometriotic tissues as compared to healthy tissues.

## Discussion

The present study was performed with the aim of validating an animal model in order to gain deeper insights into the development of endometriosis (Taniguchi et al. 2021). Since the normalization of hypoxia may be a potential target in the development of new therapies for the treatment of endometriosis, our goal was to confirm if it would be possible to reliably evaluate the level of hypoxia in endometriotic lesions in the chosen murine model of endometriosis. Such experiments require autologous and/or syngeneic implantation of tissues to allow for the development of lesions mimicking endometriosis, principally permitting angiogenesis development and establishing a link to hypoxia-dependent signals during the course of the disease (Berg et al. 2016; deSouza et al. 2022) to help decipher the molecular mechanism of modulation (Vashisht et al. 2020). The ovaries remained intact in order to properly recreate all the processes occurring during endometriosis and to maintain the hypothalamic-pituitary-ovarian axis. Preserving oestrogen production, which is a hallmark of endometriosis development, allows the potential drug effect during a normal hormonal cycle to be evaluated (Burns et al. 2022). The mouse model displays characteristics that are similar to human lesions in terms of cell proliferation, neovascularization, and epithelial-stromal tissue composition (Zhao et al. 2014). The lesions sutured to the peritoneal cavity led to the establishment of cystic endometriotic-like lesions filled with fluid. In this work, using ultrasonography designed for small animal imaging, we observed the growth of implanted tissues throughout the three weeks of development (Moran et al. 2022). This live, non-invasive method of visualization enables the observation of the evolution of lesions throughout the experiment, which is fundamental for further preclinical studies of any type of treatment. This also permits a precise analysis of the size, volume, and shape evolution, as well as the quantitative measurement of lesions from the state of implantation to the invasion. In this study, on days 10 and 21 of the experiment, the ultrasound imaging of the lesion showed that the mean 3D volume of the developed lesions was 4.87 mm^3^ and 10.70 mm^3^, respectively. A histological analysis of the lesions revealed that they contain large fluid-filled cysts which are characteristic of human endometriotic lesions (Vashisht et al. 2020). The colour Doppler mode confirmed the presence of small blood vessels around the endometriotic-like lesions, which were also observed during tissue collection. Moreover, the Doppler mode, which is used to evaluate the blood flow direction and intensity in hypoxic lesions, allowed blood flow establishment to be monitored alongside the development of the disease, tracking vessel growth and their blood-carrying capacity to the pathologic lesion (Collet et al. 2016; Szade et al. 2016). The monitoring of blood flow and its ability to transport oxygen is essential in all hypoxia-dependent diseases in which the evolution of the pathology depends on the establishment of hypoxia and its maintenance due to the so-called pathological angiogenesis being induced. Hypoxia plays an important role in the development of endometriosis wherein the growth of new blood vessels is essential. An inadequate transport of oxygen to the tissues changes the microenvironment in terms of immune cell recruitment and action, phenotypic adaptation, and conduciveness to endometrial lesion growth (Maksym et al. 2021). Although it has been demonstrated that the inhibition of HIF-1α expression and, in consequence, angiogenesis, suppress the growth of lesions (Becker et al. 2008), it seems that vascular normalization may provide many more benefits. Attempts to change the structure and efficacy of the vessels may lead to a real change in the composition of the microenvironment that will permit a more successful application of treatments, avoiding the challenging pitfalls resulting from the inaccessibility caused by the non-flowing blood in the pathological vessels. This is one of the general features of hypoxia-dependent pathologies, emphasising the importance of vessel normalization in the course of treatment (Choi and Jung 2023; Sun et al. 2023). In line with the hypothesis that, in this model, hypoxia does develop as it does in a human healthy endometrium during the given phases of menstruation, but that it does so chronically in a pathological endometrium, we first validated if hypoxia is indeed established in the induced endometriotic lesions. Using EF5, a pentafluorinated derivative of the 2-nitroimidazole, which is metabolically reduced by oxygen-inhibitable nitroreductase(s), we confirmed that hypoxia did develop in this model and was present in all the examined tissues. The existence and function of a hypoxia gradient in a healthy endometrium or during menstruation has been previously observed in mouse models using a pimonidazole derivative (Zhang et al. 2022; Cousins et al. 2016). We show here for the first time that the reduction activity of EF5 differed between the epithelial and stromal cells in the ectopic endometrium. EF5 labelling was intensely positive in the epithelial layer vs the stromal cells in the endometriotic lesions and in the uteri from mice with endometriosis as compared to the uteri from control mice. A previous study by Liu et al. (Liu et al. 2018) showed immunohistochemical staining of the hypoxia marker HIF-1α in normal, eutopic, and ectopic endometria from women with endometriosis, where the transcription-active HIF-1α was indeed predominantly located in the nuclei of epithelial and stromal cells. Here, we present a decrease in tissue oxygenation and show that hypoxia is distributed differently throughout the cell layers. This data has been supported by a direct follow-up of the pO_2_ value measured by the oxygen-dependent quenching of fluorescence emitted by platinum-based dye, which is reported using OxyLite, the gold standard for oxygenation studies (Wei et al. 2019). Although direct measurement was not possible in the live animals due to the size of the lesions, the oxygen tension was measured in the lesions and the uteri extemporaneously, after the sacrifice of the animals. This showed a reduction of the oxygen tension in the cyst (50%) and wall (83%) of the lesion compared to that in a normal uterus. To find out whether hypoxia-targeting strategies accompanied by vessel normalization are a suitable approach for future treatment protocols, an assessment of the main genes implicated in hypoxia-mediated vessel formation, like *Hif1a* and *Epas1* demonstrating hypoxia establishment, and *Vegfa* and *Pten* for cell response and pathological vs normal angiogenesis, was carried out in terms of their expression. At the first attempt, an expression comparison was performed on the entire tissue representing the normal vs pathologic uteri. In this study, we observed a downregulation of *Pten* expression in the uteri of mice with endometriosis, which is in line with the previously published data showing that sporadic mutation or inactivation of *Pten* was found in endometriosis or endometrial hyperplasias (Tashiro et al. 1997; Sato et al. 2000; Govatati et al. 2014; Yang et al. 2021; Yang et al. 2016). Interestingly, the profile of *Pten* expression changes in ectopic endometriotic lesions. We observed that the expression of *Pten* does not change or is upregulated in endometriotic lesions collected from the peritoneum in comparison to normal or pathologic uteri, respectively. The latter seems to contradict previously published data, however, these results indicate the importance of distinguishing between ectopic and eutopic endometria. The molecular mechanisms controlling vascularization through *Pten* were extensively reviewed by Orozco-García et al. (2023). It was shown that the *Pten* expression level changes in different types of cells involved in the angiogenesis process and that it depends on the stage of vascularization. Endothelial cells exhibiting a stalk type phenotype (located at the base of the sprout, establishing adherent/tight junctions, and forming a vascular lumen), which are widely described to be proliferative, present an increased expression of *Pten* (Orozco-García et al. 2023; Serra et al. 2015). The colour Doppler mode of analysis confirmed that vessels are being formed around the implanted endometriotic lesions, which may explain the increased expression of *Pten.* Moreover, *Mdm2*, *Hif1a*, *Epas1,* and *Vegfa* expression increased in endometriotic lesions, which confirmed the establishment of hypoxia in the endometriotic lesions and gene overexpression, as *Epas1* indicated the stability of the hypoxic state. Considering hypoxia and its role on angiogenesis in endometriosis pathology along with its decisive influence on cancer progression, angiogenesis is favoured by the strong expression of *Vegf*a despite the overexpression of *Pten*. Although *Pten* is strongly induced, the restrictive effect on *Trp53* appears to be counteracted by the high expression of *Mdm2,* the main inhibitor of *Trp53* action at protein level. The present murine model of endometriosis corroborates the previous observation of Sang et al. (2019) in tissue samples collected from patients showing that MDM2 and p53 restrain and influence each other in the pathogenesis and development of endometriosis. These genes are considered to be the most significant for the malignant evolution of endometriosis; thus, their strong modulation evidenced in this work is indicative of model reliability. Moreover, the fact that their regulatory activity is fine-tuned by their phosphorylated forms and balance in the various tissue cells should also be taken into account. This precise definition could bring key answers through the separate analysis of cross regulation in vessel endothelial cells and lesion wall epithelial cells under the influence of hypoxia.

Although presented data were obtained from experiments conducted on a small number of animals, the results are statistically significant and indicate the appropriateness of selecting this model. This pilot experiment is an important introduction to further research which now has a justification to be carried out on a larger number of animals. The idea is to investigate how pharmacological normalization of angiogenesis and alleviation of hypoxia influence the development and establishment of endometriotic lesions on the one hand. On the other hand, this will serve as a basis for enhancing the efficacy of treatments that are not able to properly reach the diseased site in the context of pathologic angiogenesis. The presented model brings the proof that it is possible to observe the effects of such therapeutic attempts at separate key steps, by non- invasive, objective preclinical validation methods provided by photoacoustic.

## Conclusions

A non-invasive approach using Vevo 3100 ultra high-frequency ultrasound was applied here for the first time to the study of endometriosis in an animal model. It proved suitable for the monitoring of endometriotic lesions in live mice and allowed the detection of lesions as early as 10 days into development. It also demonstrated that the implantation model was 100% efficient as all the mice from the induced group produced endometriotic lesions. This method brings a considerable improvement in the follow-up of endometriotic pathology because it also validates the hypoxia dependency of the disease, as reported biochemically by ex vivo immunofluorescence assessment and the measurement of hypoxia-associated genes. Moreover, the angiogenic reaction shown here can also be evidenced by the applied ultrasonographic method, which opens up entirely new avenues for elaborating new treatment strategies based on the alleviation of hypoxia and restoration of blood flow.

## Data Availability

The datasets used and/or analysed during the current study are available from the corresponding author on reasonable request.
